# A Short-Term Hybrid TCN-GRU Prediction Model of Bike-Sharing Demand Based on Travel Characteristics Mining

**DOI:** 10.3390/e24091193

**Published:** 2022-08-26

**Authors:** Shenghan Zhou, Chaofei Song, Tianhuai Wang, Xing Pan, Wenbing Chang, Linchao Yang

**Affiliations:** School of Reliability and Systems Engineering, Beihang University, Beijing 100191, China

**Keywords:** short-term demand prediction, bike-sharing, travel characteristics analysis, hybrid TCN-GRU model

## Abstract

This paper proposes an accurate short-term prediction model of bike-sharing demand with the hybrid TCN-GRU method. The emergence of shared bicycles has provided people with a low-carbon, green and healthy way of transportation. However, the explosive growth and free-form development of bike-sharing has also brought about a series of problems in the area of urban governance, creating a new opportunity and challenge in the use of a large amount of historical data for regional bike-sharing traffic flow predictions. In this study, we built an accurate short-term prediction model of bike-sharing demand with the bike-sharing dataset from 2015 to 2017 in London. First, we conducted a multidimensional bike-sharing travel characteristics analysis based on explanatory variables such as weather, temperature, and humidity. This will help us to understand the travel characteristics of local people, will facilitate traffic management and, to a certain extent, improve traffic congestion. Then, the explanatory variables that help predict the demand for bike-sharing were obtained using the Granger causality with the entropy theory-based MIC method to verify each other. The Multivariate Temporal Convolutional Network (TCN) and Gated Recurrent Unit (GRU) model were integrated to build the prediction model, and this is abbreviated as the TCN-GRU model. The fitted coefficient of determination R2 and explainable variance score (EVar) of the dataset reached 98.42% and 98.49%, respectively. Meanwhile, the mean absolute error (MAE) and root mean square error (RMSE) were at least 1.98% and 2.4% lower than those in other models. The results show that the TCN-GRU model has strong efficiency and robustness. The model can be used to make short-term accurate predictions of bike-sharing demand in the region, so as to provide decision support for intelligent dispatching and urban traffic safety improvement, which will help to promote the development of green and low-carbon mobility in the future.

## 1. Introduction

With the gradual improvement of people’s living standards and the enhancement of environmental awareness, the series of negative social impacts brought about by rapid economic growth, such as traffic congestion, environmental degradation and noise pollution caused by overloaded motor vehicle usage, have undoubtedly led to an increasing demand for green and low-carbon means of travel. Bike-sharing has not only made a contribution to low-carbon environmental protection, but also alleviated the problem of “human transportation” in the area of public transportation to a certain extent. However, the explosive growth and “free-range” development of bike-sharing has also brought about a series of problems: first, given the lack of supervision, the excessive proliferation of bike-sharing has caused a waste of resources and urban “bicycle pollution”; second, the lack of overall layout planning for bike-sharing parking has led to the occupation of crowded public land; third, the free-moving bikes are unevenly distributed in time and space, and their operation and maintenance is not timely.

Building a prediction model based on the historical data of bike-sharing demand can effectively explain the time series characteristics of this phenomenon, but the influence of other elements in the bike-sharing system is not considered; thus, there is a certain one-sidedness, and a limit to the ability to explain and predict the fluctuation mechanism of bicycle travel demand [1]. Related studies have found that factors affecting the demand for bike-sharing rides include external factors such as weather, air quality, spatial location, user price sensitivity, and chance events, in addition to historical data on travel demand [2]. Through a survey of bike-sharing programs in Beijing, Campbell et al. [3] pointed out that the main factors affecting the demand for bike-sharing are distance, temperature, precipitation, and air quality, and that the users’ own demographic characteristics (including income, gender, and occupation) have no significant effect on the demand for bicycles. Matton et al. [4] pointed out that climatic conditions such as temperature, wind, and precipitation are the main factors affecting the demand for bike-sharing, and Faghih et al. [5] suggested that point-in-time factors are also important variables affecting the demand for bike-sharing, including the time of day, whether it is a weekend, and peak hours. In addition, weather factors and point-in-time factors [6,7,8], population density [9,10], the availability of bicycle lane facilities [10,11,12], and distance to the urban CBD and universities [5,10,13] are also related to the demand for bike-sharing.

Therefore, some studies have started to incorporate external factors such as weather, time factors and holiday factors into the independent variables of bike-sharing demand prediction. Li et al. [14] established an LSTM linear regression model considering the distance variable of users’ rides, and the results of the study show that the prediction accuracy was improved compared with the existing time series prediction models. Li et al. [15] proposed a prediction method based on a clustering algorithm with an augmented regression tree model based on weather conditions, temperature, and wind speed, so as to predict the number of rentals and returns of bicycles at stations separately. Chen et al. [16] argued that the demand for bike-sharing is affected not only by general factors such as time and weather, but also by contingent factors such as traffic events, and proposed a dynamic cluster-based forecasting framework.

From the perspective of forecasting model development, statistical methods such as the Autoregressive Integrated Moving Average model (ARIMA) were first applied to solve the bike-sharing cycling demand forecasting problem. Statistical inferential forecasting methods based on statistics include traditional models such as ARIMA models, regression analysis and Markov chains [17]. Andreas et al. [18] developed a prediction model based on a differential sliding average autoregressive model, using operational data from bicycle companies and data from bike-sharing in the Barcelona community, to forecast the number of available bicycles at each bicycle station. To investigate the characteristics and patterns of peak bicycle demand hours, Lin et al. built an ARIMA model [19]. Yan et al. [20] considered both the temporal and spatial dependence of bicycle borrowing and returning demand. For the time series, the cyclicality and trend of bicycle travel demand were obtained by building an ARIMA model considering seasonal patterns; for the spatio-temporal dependence, the inter-cluster transfer characteristics were portrayed by building a Bayesian transfer network model. Zhou et al. proposed a prediction method based on the Markov chain model. The study evaluated the model using data from the public bicycle system in Zhongshan City. The results of the case analysis verify the high prediction accuracy and generalization ability of the Markov chain model [21].

The traditional statistical methods are more sensitive to data, and the presence of data noise can greatly reduce the reliability of model parameter estimation. At the same time, there is a certain degree of spatial and temporal dependence between the demand for bike-sharing trips and external influences such as weather, and the prediction models based on statistical methods have weak explanatory power for the complex nonlinear relationships between bicycle demand and the influencing factors. In the era of big data, nonparametric methods can handle massive traffic trip data and discover the dynamic characteristics of the bicycle system.

Nonparametric methods include machine learning methods and deep learning methods. Using machine learning methods such as random forests [22], Bayesian networks [23], GBDT [24] and artificial neural networks (ANN) [25], nonlinear prediction models can be built based using a large amount of bike-share historical travel data to predict future bike-share demand at any time interval. In addition, deep learning methods are gradually being used to predict short-term bikeshare demand. Wang et al. [26] used a long short-term memory (LSTM) neural network and gated recursive units (GRU) to predict short-term bicycle availability. Chen et al. [27] proposed a recurrent neural network (RNN) using time, weather, and seasonal data to predict the rental and return demand for each station in the system. Zhang et al. [28] proposed a deep learning model for the short-term prediction of bike-sharing demand, considering the correlation between bike-sharing users and public transportation riders. He et al. [29] proposed a bike-share demand prediction (BDP) model that incorporates a temporal convolutional network (TCN) and a self-attention mechanism. The BDP model extracts feature information with multiple inputs of multiple sources of data, and uses the parallelism of the self-attention mechanism to improve the training speed. A better prediction accuracy is obtained in comparison with other models. Ma et al. [30] proposed a Spatio-Temporal Graphical Attention Long-Term Memory (STGA-LSTM) neural network framework for predicting demand for bike-sharing at the station level using a multi-source dataset. This short-term prediction model can be used to help bike-sharing users make better route choices, and help operators implement dynamic redistribution strategies. Mehdizadeh et al. [31] proposed a hybrid CNN-LSTM model for the short-term prediction of mountain biking demand, which had considerable prediction accuracy during the COVID-19 pandemic after adding additional variables such as weather conditions and time of day.

The research for this thesis includes two main aspects: (1) mining the travel pattern of bike-sharing users, analyzing the travel characteristics of residents, and providing references for bicycle demand prediction; (2) making accurate predictions of bike-sharing demand, improving the bicycle turnover rate, and providing a decision basis for the intelligent scheduling of regional bike-sharing.

The study is divided into the following sections: Section 1 focuses on the study background, study content and literature review. Section 2 mainly concerns data description and pre-processing, including a preliminary correlation analysis. Section 3 mines the bike-sharing trip characteristics through multiple dimensions, such as time, temperature, humidity, and weather. Section 4 introduces the TCN model, MIC variable selection method, GRU model, hybrid time series model and evaluation indicators. This is followed by multiple rounds of comparison experiments for validation. Section 5 and Section 6 are the discussion and conclusions sections, respectively.

## 2. Data Overview and Preprocessing

### 2.1. Data Overview

This paper used the London bike-sharing public dataset as the subject of the study. The dataset recorded a total of 17,414 data points (one data point generated every hour, i.e., 24 data points per day) for the London area from 4 January 2015 to 3 January 2017. The dataset recorded the influencing factors, such as weather and travel time, related to the demand of bike-sharing; we performed a data background gain by adding data nouns such as “Hour” and “Month” with timestamp information. The descriptions of the data terms and examples are shown in Table 1.

### 2.2. Data Preprocessing

Since the dimensionality and magnitude of each variable are not uniform, to eliminate the influence of magnitude and to speed up model training, the normalization method was used to normalize the data. This involves a linear transformation of the original data that maps the data values to the [0, 1] interval. The transformation function is shown in Equation (1):

(1) $$x^{*}=\frac{x-min}{\max-min}$$

where max is the maximum value of the data, and min is the minimum value.

### 2.3. Correlation Analysis

There is correlation between different features in the data, resulting in feature redundancy. In addition, not all influencing factors are related to the demand for bike-sharing. The correlation analysis aimed to investigate the correlation between bike-sharing variables, i.e., a preliminary analysis of other variables that are correlated with the demand for bike-sharing. After the normality test, the data of most of the variables used in this study did not conform to a normal distribution. Therefore, we used Spearman’s rank correlation coefficient for measuring the linear correlation between the variables [32].

The rank is the average descending position of a number in the overall data. If X and Y are two observed variables with sample size n, and for each sample ($X_{i}$, $Y_{i}$), the corresponding rank is ($x_{i}$,$y_{i}$), then the Spearman’s rank correlation coefficient ρ between these two variables is determined via Equation (2).

(2) $$\rho =1-\frac{6\sum {\left(x_{i}-y_{i}\right)}^{2}}{n\left(n^{2}-1\right)}$$

The Spearman’s rank correlation coefficient ranges within [−1, 1]. When the absolute value is close to 1, this indicates that the two variables are more strongly correlated. When the value is positive, if one of the two characteristics shows an increasing trend, the other also tends to increase, and when the value is 1, it indicates a perfect positive correlation; when the value is negative, if one of the two characteristics tends to increase, the other tends to decrease, and when the value is −1, it indicates a perfect negative correlation; when the value is 0, this indicates a perfect non-correlation (the tendency of one to change does not change with that of the other). In general, the absolute value of the correlation coefficient in the range of (0.8, 1.0) is considered as very strong correlation, while the range (0.6, 0.8) is considered strong correlation, (0.4,0.6) moderate correlation, (0.2, 0.4) weak correlation, and (0, 0.2) very weak or no correlation.

**Figure 1:**
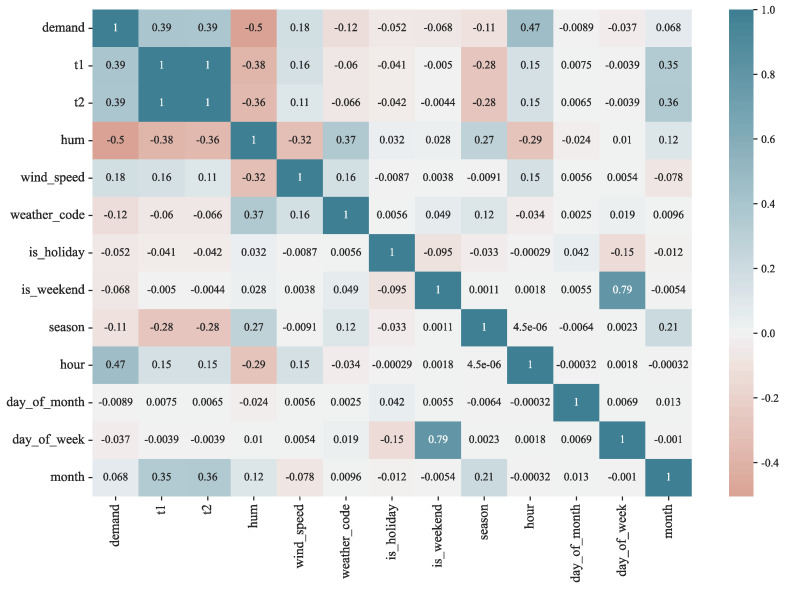
Bike-sharing demand correlation analysis heat map.

The results of the correlation analysis between demand and each variable are shown in Figure 1, which shows that the actual temperature t1 is highly correlated with the subjectively perceived temperature t2, and there is a problem of feature redundancy. In addition, temperature demand shows a weak positive correlation with temperature, while demand shows a moderate negative correlation with humidity, a very weak positive correlation with temperature, and a very weak negative correlation with weather and season. The correlation analysis can roughly determine the linear relationship between demand and its influencing factors. In order to obtain the trend of demand under its different influencing factors, data mining methods can be used to analyze the travel characteristics of bike-sharing.

## 3. Bike-Sharing Travel Characteristics Analysis

As an important means of transportation for urban residents, bike-sharing often presents different characteristics due to a variety of factors, which must be explored for the purpose of traffic management. Therefore, based on the considered dataset, we explored bike-sharing travel characteristics via several dimensions such as time, temperature, humidity, and weather [33].

### 3.1. Bike-Sharing Travel: Time Characteristics Analysis

#### 3.1.1. Demand Varies with the Hours and Months

**Figure 2:**
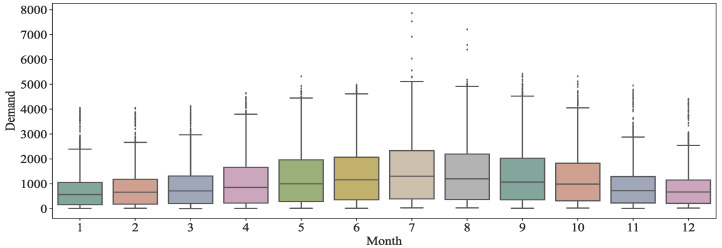
Box plot of demand for bike-sharing in different months.

First, we assessed the distribution of the demand for bike-sharing in different months, and the results are shown in Figure 2. The demand shows an obvious single hump shape that develops with the month, i.e., the demand for bike-sharing in the area gradually increases from January until it peaks in July, and it then starts to decrease month by month.

**Figure 3:**
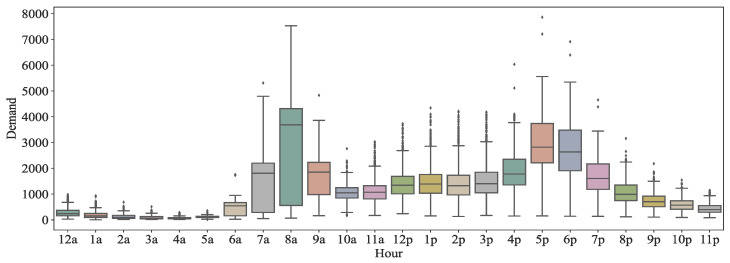
Box plot of demand for bike-sharing at different hours.

Next, we determined the distribution of bike-sharing demand at different times of the day, and the results are shown in Figure 3. The demand shows an obvious double-hump shape that develops with the time of the day, that is, the demand for bike-sharing in the area is high at 7 and 8 a.m. and 5 and 6 p.m. This result coincides perfectly with people’s commuting time to and from work on weekdays, and also reflects that bike-sharing is in the highest demand when people commute to and from work, suggesting that bike-sharing can provide convenience for people’s work travel.

**Figure 4:**
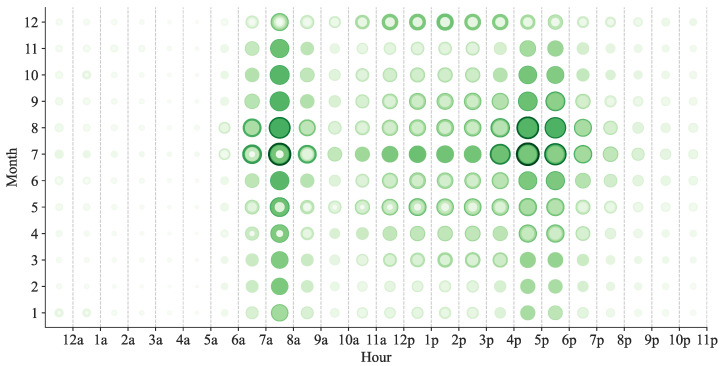
Time bubble map of bike-sharing demand.

We analyzed the distribution of bike-sharing demand by month at different moments of the day with bubble chart statistics, where in larger bubbles indicate higher demand. The statistical results are shown in Figure 4. As can be seen, the vast majority of months show a double-hump distribution of demand. However, in December, demand for shared bikes increases when people are at work, while demand is roughly the same throughout the afternoon from 12:00 to 6:00, with no clear trend. This may have more to do with the local climate as well as holidays.

**Figure 5:**
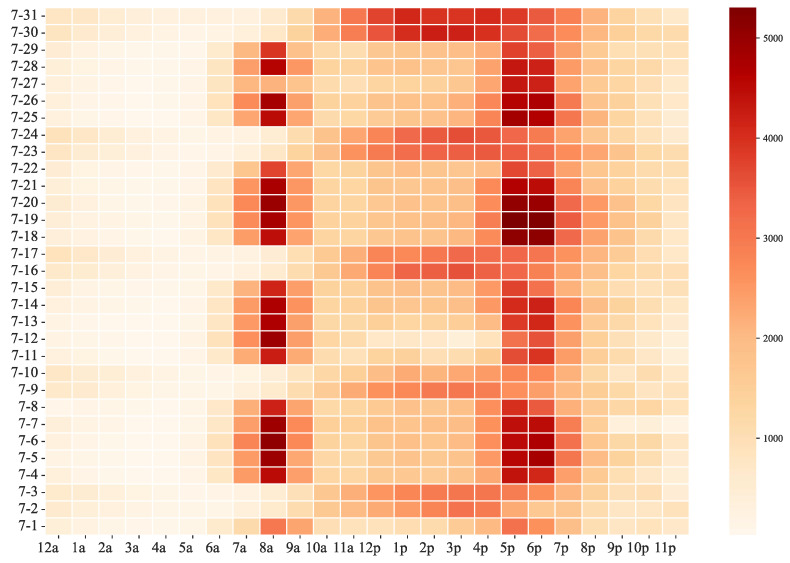
Bike-sharing demand time heat map.

It is known from the previous analysis that July is the month with the highest demand for bike-sharing; so, we took July 2016 as the research object and analyzed the daily demand changes in this month using heat maps, and the statistical results are shown in Figure 5. It can be seen that there is an obvious cycle pattern in the demand distribution, with every seven days being a cycle, and the demand distribution on five of the days corresponds to the weekday travel characteristics, i.e., the obvious double-hump feature of on and off work. This also reflects the obvious difference in the distribution of demand on weekdays and non-working days. There is a high demand for bike-sharing on July 30 and 31, which may be related to the local Prudential Ride London event, a popular ride that locals say turned London into a bicycle-centric environment.

#### 3.1.2. Demand Varies with Working and Nonworking Days

**Figure 6:**
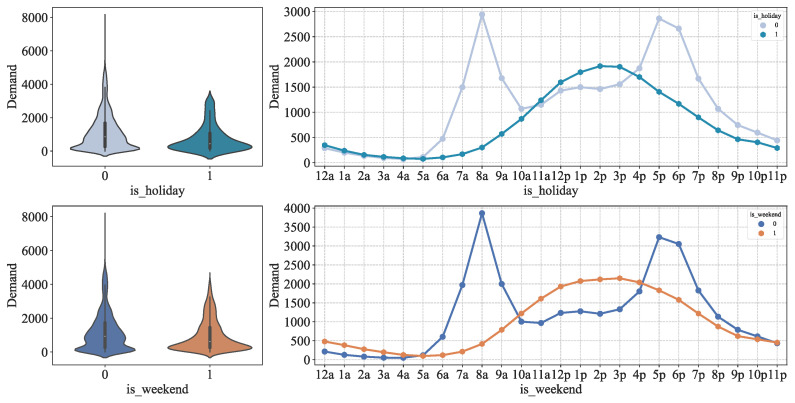
Bike-sharing demand on working and nonworking days.

We found in the previous analysis that there is a significant difference in the distribution of bike-sharing demand between weekdays and non-weekdays. Therefore, we took weekends and holidays as the research object and used weekday data for comparative analysis, and the analysis results are shown in Figure 6. It can be seen that the distribution of people’s travel characteristics on holidays and weekends is roughly the same. On weekdays, 8:00 and 17:00 and 18:00 are the peak times for car use, which coincides with the time points for going to and leaving work. In the case of nonworking days 14:00–15:00 is the real peak period of car usage. This reflects people’s preference for using shared bikes to travel in the afternoon during nonworking days.

#### 3.1.3. Demand Varies with the Season

**Figure 7:**
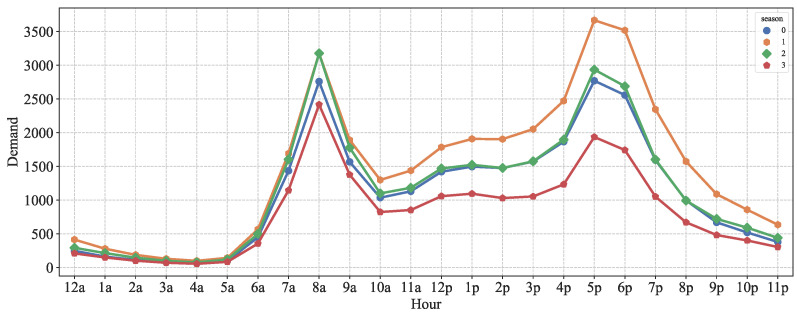
Bike-sharing demand in different seasons.

In addition, we analyzed the distribution of bike-sharing demand by season at different moments of the day through line graph statistics. The results are shown in Figure 7. It can be seen that the trend of bike-sharing demand is more or less the same in different seasons, with higher demand in summer and autumn, and the lowest in winter, which is obviously related to the seasonal climate.

### 3.2. Bike-Sharing Travel: Meteorology Characteristics Analysis

**Figure 8:**
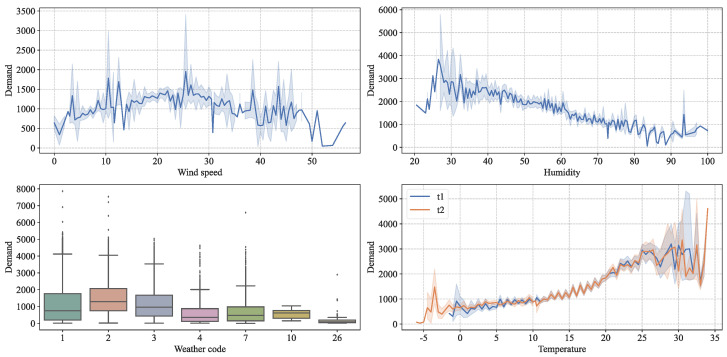
Bike-sharing demand under different meteorological conditions.

Many studies have shown that, as external environmental factors, weather type [34], temperature [35], and air quality [36], also have direct and indirect effects on travel characteristics. To investigate the influence of weather characteristics on bike-sharing trips, we obtained the trends of bike-sharing demand with wind speed, humidity, weather type, and temperature using line plots as well as box plots. The results are shown in Figure 8. It can be seen that there is a local peak at a wind speed of 25 km/h, and the demand decreases at higher and lower wind speeds. There is a negative correlation between air humidity and demand; that is, with greater air humidity, the overall demand shows a decreasing trend. In weather codes 2 and 3, that is, when the weather type is either less cloudy or cloudy, the demand is larger; when the weather is more severe, the demand gradually decreases, and when the weather code is 26 (snow), the demand is almost 0. The demand shows a trend of increasing first and then decreasing with the rise in temperature; that is, below the temperature is 25 °C, the demand shows a relatively strong positive correlation with temperature, and after the temperature exceeds 25 °C, the demand shows a relatively weak negative correlation with temperature.

### 3.3. Bike-Sharing Travel: Characteristics Analysis Based on Granger Causality Test

The correlation analysis lacks an explanation for the causal mechanism of the fluctuation in bike-sharing demand, and we next explore the impact of weather and other characteristics on the demand for bike-sharing travel from the causality perspective. Weather data indicators include t1, hum, weather_code and wind_speed. In order to further screen the indicators that help predict the demand for bike-sharing travel, this paper uses the Granger causality test method for weather and other features’ screening. The basic idea of the method is that [37], if a series X helps to explain the future trend of series Y—that is, in the regression model of series Y regarding its own historical information, adding the historical information of X will significantly improve the explanatory power of the regression model—then series X is the Granger cause of series Y.

Before Granger causality tests were performed on the weather indicator grid, the unit root method was used to perform a smoothness test. For non-stationary series, differencing was performed until it passed the stationarity test. The results of the causality test for each variable at the significance level α=0.05 are presented in Table 2.

When p<0.05 rejects the original hypothesis, this indicates that there is a Granger causality with statistical significance between weather indicators t1, hum and the demand for bike-sharing, i.e., adding weather indicators t1 and hum to the model helps predict the demand.

The analysis of bike-sharing travel characteristics in London reveals that both point-in-time factors [5] and weather conditions [4] affect the variation in bike-sharing demand to varying degrees. There is consistency and interoperability between our analysis and the results of other literature analyses. In addition, we found that the factors influencing bike-sharing demand were roughly the same across regions, i.e., differences in regional attributes, culture, climate, and ethnicity do not affect travel characteristics. A survey of the Beijing [3] bike-sharing program also found that users’ own demographic characteristics do not have a significant effect on bicycle demand.

## 4. Bike-Sharing Short-Term Demand Prediction

The bike-sharing demand data are susceptible to the influence of time, climate and traffic management policies, showing strong volatility and nonlinearity. The bike-sharing demand data used in this paper are hourly, and the sample size is relatively small. The deep neural network has a strong fitting ability for nonlinear data but is prone to the risk of overfitting in the case of small samples. Based on the above analysis, this paper has tried to combine the typical models of deep learning temporal prediction, GRU and TCN, with the principle of the least-squared error sum. In so doing we aimed to reduce the possibility of overfitting and to take advantage of the fitting of deep learning models on nonlinear and non-stationary data, in order to improve the prediction ability of the models.

### 4.1. Temporal Convolutional Network (TCN)

TCN is a novel architecture based on a Convolutional Neural Network (CNN). Unlike general CNNs, TCNs use structures such as expanded causal convolution and residual blocks [38,39,40]. This gives them the ability to extract features and achieve prediction from large sample time series, and TCNs can effectively address the performance degradation of deep networks during network training. TCN consists of dilated, causal 1D fully convolutional layers with the same input and output lengths. The convolution in the TCN model is causal convolution, wherein the layers are causally related to each other, thus ensuring that no historical information or future data will be missed. In addition, TCN can map sequences of arbitrary length to output sequences of the same length, using residual modules and dilation convolution to better control the memory length of the model and improve the predictive power.

#### 4.1.1. TCN Modeling

Supposing that the input sequence is given as $\left\{x_{1},x_{2},\cdots ,x_{t}\right\}$, and the expected predicted output is $\left\{{\hat{y}}_{1},{\hat{y}}_{2},\cdots ,{\hat{y}}_{t}\right\}$, the equation of the predicted output versus the input sequence can be presented by Equation (3):

(3) $$\left({\hat{y}}_{1},{\hat{y}}_{2},\cdots ,{\hat{y}}_{t}\right)=f\left(x_{1},x_{2},\cdots ,x_{t}\right)$$

where ${\hat{y}}_{t}$ is only related to the input sequence at time t and in the past, and is independent of any future input. The purpose of TCN modeling is to establish a mapping relationship f between the input and output sequences, and its objective function is to minimize the error loss between the actual output $\left\{y_{1},y_{2},\cdots ,y_{t}\right\}$ and the predicted values $\left\{{\hat{y}}_{1},{\hat{y}}_{2},\cdots ,{\hat{y}}_{t}\right\}$.

#### 4.1.2. Extended Causal Convolution

The causal convolutions were originally proposed in the WaveNets [41] networks for learning the input audio data before moment t to predict the output at moment t+1. Compared to RNNs, no circular connections are used in models using causal convolutions, so time series data can be input in parallel, which allows for faster network training, especially for large-sample time series [42]. However, standard causal convolution requires increasing the receptive field of neurons in the neural network by stacking many network layers or using very large convolutional kernels when dealing with large sample time series. For this reason, TCN uses the Dilated Causal Convolution (DCC) technique to achieve an increase in the perceptual field without a significant increase in computational cost. DCC is a convolution operation that performs a step-skipping operation on the input sequence, and its expression is given by Equation (4):

(4) $$F\left(i\right)=\overset{k}{\underset{j=1}{\sum }}h\left(j\right)x\left(i-dj\right)$$

where F(i) is the convolution result for the ith element in the sequence $\left\{x_{1},x_{2},\cdots ,x_{t}\right\}$; h(j) is the convolution kernel, and for a one-dimensional sequence its convolution kernel size K=1×k; d is the expansion factor (when d=1, that is the standard causal convolution).

**Figure 9:**
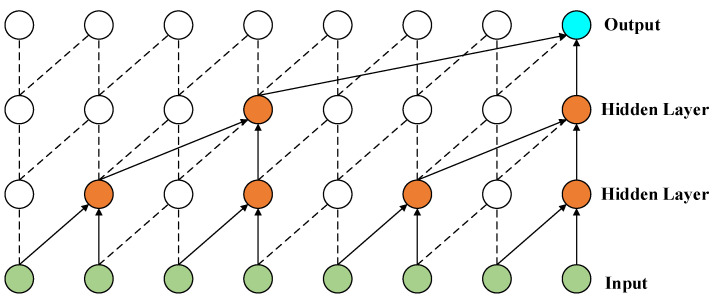
Schematic diagram of extended causal convolution.

The structure of DCC is shown in Figure 9 (K=1×2 and $d=2^{l}-1$, l is the number of hidden layers). Compared with standard causal convolution, DCC allows the output to be associated with as many inputs as possible with the same number of network layers. Multilayer stacking combined with extended causal convolution also allows deep learning networks to achieve very large sensory fields with fewer network layers [43]. Moreover, the sliding operation of the convolution kernel on the input data allows the TCN to handle inputs of variable length. Thus, in conjunction with the updating of the model’s input data (i.e., the predicted values from the previous moment are added to the input as information), new predictions can be continuously computed and output.

#### 4.1.3. Residual Block

Residual Block (RB) is proposed to solve the degradation problem of deep learning networks, and its core idea is to introduce a “jump connection” operation that skips one or more layers [44]. Assuming that x is the input of the residual block, the output o of the residual block is shown in Equation (5), which is the result of linear variation and mapping through the activation function. Since the residual κ(x) will not be zero in practice, the stacked layers in the deep learning network can always learn new features, so the learning performance of the deep network will not degrade [45].

In TCN modeling, using a network structure combining RB and DCC can effectively improve the feature learning capability and robustness of TCN models.

(5) o=Activation(x+κ(x))

### 4.2. Gated Recurrent Unit (GRU)

**Figure 10:**
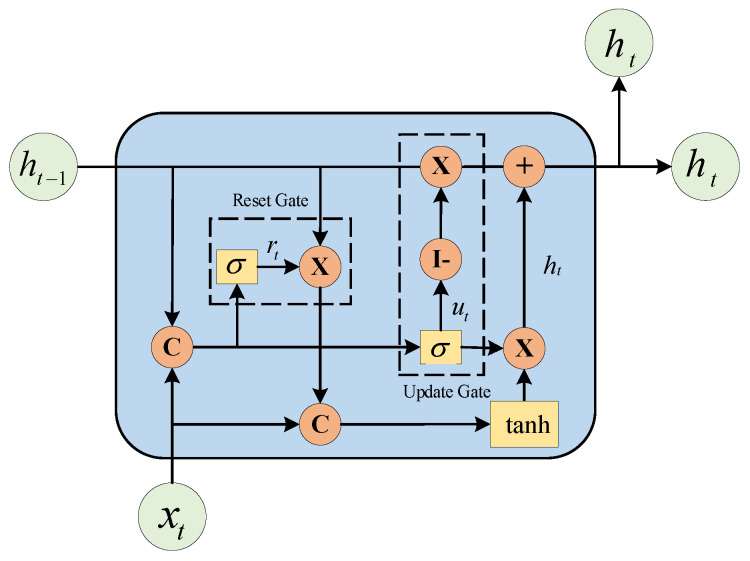
GRU model’ internal structure.

LSTM [46] and GRU [47] show strong potential applicability in the data prediction problem studied in this paper, with GRU performing slightly better. Compared with the LSTM method, GRU requires fewer training parameters, is easier to converge and can reduce the risk of model overfitting in the case of limited time series data. GRU optimizes the three gate functions of LSTM, turning the set of forgetting gates and input gates into a single update gate, and mixing the neuron states with the hidden states. This can effectively alleviate the problem of “gradient disappearance” in RNN networks and reduce the number of parameters of LSTM network units, shortening the training time of the model. The basic structure is shown in Figure 10, and the mathematical description is shown in Equations (6)–(10):

(6) $$r_{t}=\sigma \left(W_{r}\cdot \left[h_{t-1},x_{t}\right]\right)$$

(7) $$u_{t}=\sigma \left(W_{u}\cdot \left[h_{t-1},x_{t}\right]\right)$$

(8) $$\tilde{h_{t}}=\tanh(W_{\tilde{h}}\cdot \left[r_{t}*h_{t-1},x_{t}\right])$$

(9) $$h_{t}=\left(1-u_{t}\right)*h_{t-1}+u_{t}*\tilde{h_{t}}$$

(10) $$y_{t}=\sigma \left(W_{o}\cdot h_{t}\right)$$

where $x_{t}$, $h_{t-1}$, $h_{t}$, $r_{t}$, $u_{t}$, $\tilde{h_{t}}$ and $y_{t}$ are the input vector, the state memory variable of the previous moment, the state memory variable of the current moment, the state of the update gate, the state of the reset gate, the state of the current candidate set, and the output vector of the current moment, respectively. $W_{r}$, $W_{u}$, $W_{\tilde{h}}$ and $W_{o}$ are the weight parameters used for multiplying the update gate, reset gate, candidate set, and output vector with the connection matrix composed of $x_{t}$ and $h_{t-1}$, respectively; I denotes unit matrix; ⋅ denotes the matrix dot product; ∗ denotes the matrix product; and σ denotes the sigmoid activation function.

GRU uses update and reset gates as core modules. The splicing matrix of the input variable $x_{t}$ and the state memory variable $h_{t-1}$ of the previous moment, are input into the update gate after sigmoid nonlinear transformation, which determines the extent to which the state variable of the previous moment is brought into the current state. The reset gate controls the amount of information that was written to the candidate set at the previous moment, stores the information at the previous moment by $I-u_{t}$ times $h_{t-1}$, records the information at the current moment by $u_{t}$ times $\tilde{h_{t}}$, and sums the two as the output of the current moment.

### 4.3. Hybrid Multivariate Bike-Sharing Demand Prediction Model

Hybrid model forecasting is used to try to combine different forecasting models and the information they provide to derive a hybrid forecasting model in the form of an appropriate weighted average. The key to hybrid model forecasting is how to find out the weighting coefficients, which makes the hybrid forecasting model more effective in improving the forecasting accuracy.

Different forecasting models have their own strengths, and a better linear hybrid forecasting model can be obtained by the linear combination of different forecasting models. The linear hybrid forecasting model’s form is shown in Equation (11):

(11) $${\hat{y}}_{t}=\overset{m}{\underset{i=1}{\sum }}\omega_{i}y_{i\left(t\right)}$$

(12) $$\{\begin{array}{c} \omega_{1}+\omega_{2}+\cdots +\omega_{m}=1\\ \omega_{i}\geq 0 \end{array}$$

where ${\hat{y}}_{t}$ is the combined forecast value at moment t; $y_{i\left(t\right)}$ is the forecast value of the ith forecast model at moment t; $W={\left(\omega_{1},\omega_{2},\cdots ,\omega_{m}\right)}^{T}$ is the weighting coefficient of the linear combination of m forecast models and satisfies the requirement, as shown in Equation (12).

The key to the linear combination prediction model is to determine a reasonable number of weights $\omega_{i}$, based on the principle of the minimum sum of squares of errors (SSE) [48], which can make the prediction model more effective and accurate.

(13) $$SSE=\overset{n}{\underset{t=1}{\sum }}e_{t}{}^{2}=\overset{n}{\underset{t=1}{\sum }}{\left(\overset{m}{\underset{i=1}{\sum }}\omega_{i}e_{it}\right)}^{2}=W^{T}EW$$

(14) $$\{\begin{array}{c} \min SSE=W^{T}EW\\ s.t.R_{m}W=1,W\geq 0 \end{array}$$

(15) $$W_{0}=\frac{E^{-1}R_{m}T}{R_{m}E^{-1}R_{m}T}$$

where, $e_{it}=y_{\left(t\right)}-y_{i\left(t\right)}$ denotes the forecast error of the ith forecast model at moment t; $y_{\left(t\right)}$ is a sequence of actual values of a certain index of a forecast object; $e_{t}=y_{\left(t\right)}-{\hat{y}}_{t}$ denotes the forecast error of the linear combination model at moment t; $E={\left(e_{it}\right)}_{m\times n}{\left(e_{it}\right)}^{T}{}_{m\times n}$ is the information error matrix; the optimal weighting coefficient $W_{0}$ is obtained by solving the optimal solution of the linear programming problem, where $R_{m}$ is an m-dimensional row vector with all elements of 1, and the guaranteed non-negative optimal weighting coefficients enable the linear combinatorial model to effectively improve the prediction accuracy.

**Figure 11:**
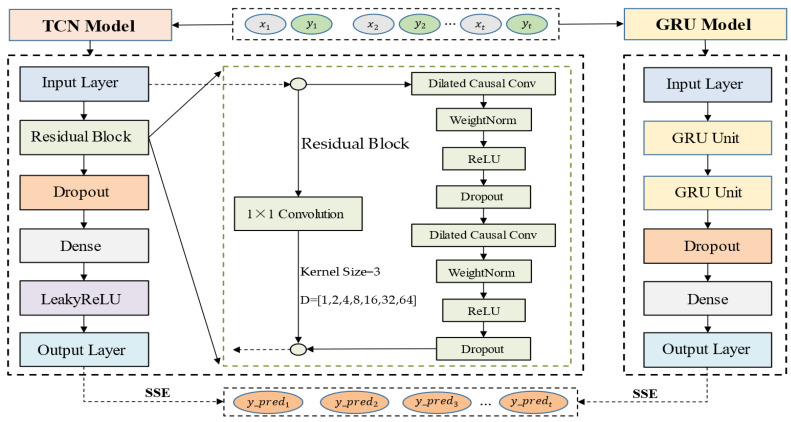
Basic structure of bike-sharing demand prediction combination model.

Our hybrid multivariate bike-sharing demand forecasting model based on the principle of minimum error sum of squares is shown in Figure 11.

### 4.4. Variables Selection

The entropy of the variables in the data set will have a direct impact on the prediction model, and this paper uses the maximum information coefficient (MIC) [49] method based on entropy theory for variable selection. MIC is a combination of information theory and probability [50] based on mutual information, and is used to detect nonlinear correlations between different variables and eventually obtain a measure of the strength of dependencies between variables. The maximum information coefficient achieves universality and equilibrium, where universality, with the help of MIC, can discover functional and nonfunctional relationships between variables; equilibrium, with the help of MIC, can be used to compare the strength of relationships between different variables, both horizontally and vertically.

Suppose that, in the data set D, the sample size is s, where an explanatory variable $X=\left\{x_{i},i=1,2,\cdots ,s\right\}$ and the explanatory variable $Y=\left\{y_{i},i=1,2,\cdots ,s\right\}$; the MIC(X,Y) between these two variables is calculated as follows.

(1) Calculate the mutual information MI(X,Y) between the explanatory variable X and the explained variable Y:
  (16) $$MIC(X,Y)=\underset{y_{i}\in Y}{\sum }\underset{x_{i}\in X}{\sum }p\left(x_{i},y_{i}\right)\log\frac{p\left(x_{i},y_{i}\right)}{p\left(x_{i}\right)p\left(y_{i}\right)}$$
  where $p\left(x_{i},y_{i}\right)$ is the joint density function of the variables X and Y. $p\left(x_{i}\right)$ is the marginal probability density function of the explanatory variable X, and $p\left(y_{i}\right)$ is the marginal probability density function of the explanatory variable Y.
(2) The variables X and Y are divided into a grid of m∗n defined as G=(m,n). To obtain the grid division that maximizes the MI, the value of MI is normalized. This normalized maximum MI can be expressed as follows:
  (17) $$MI_{D|G}\left(X,Y\right)=\frac{MI^{*}{}_{D|G}\left(X,Y\right)}{log_{min}\left\{m,n\right\}}$$
  where $MI^{*}{}_{D|G}\left(X,Y\right)$ is the maximum MI of data set D under grid G.
(3) The MIC is defined as the maximum MI under all grids G, calculated as follows:
  (18) $$\{\begin{array}{c} MIC\left(X,Y\right)=\underset{m*n<B\left(s\right)}{max}\left\{MI_{D|G}\left(X,Y\right)\right\}\\ B\left(s\right)=s^{0.6} \end{array}$$
  where B(s) is the maximum number of unit grids as a function of the number of samples.

The larger the value of MIC(X,Y), the stronger the correlation between variables X and Y. Therefore, we calculate the MIC values between all explanatory and explained variables, and select the characteristics according to Equation (19):

(19) MIC(X,Y)≥δ

where δ is the lowest variable selection threshold.

### 4.5. Model Evaluation Methods

To validate and compare the accuracy as well as the robustness of the models, we used $R^{2}$, EVar, MAE, MedAE, and RMSE as evaluation metrics, respectively.

(1) Coefficient of determination (R2)

The coefficient of determination characterizes the extent to which the regression model explains the variation in the dependent variable, or the goodness of fit of the model to the observations.

(20) $$R^{2}=1-\frac{\sum_{i=1}^{N}{\left(y_{i}-{\hat{y}}_{i}\right)}^{2}}{\sum_{i=1}^{N}{\left(y_{i}-{\bar{y}}_{i}\right)}^{2}}$$

Here, $y_{i}$ is the actual value of the ith data point; ${\hat{y}}_{i}$ is the corresponding predicted value; and ${\bar{y}}_{i}$ is the mean value of the time series. In general, the value of the coefficient of determination $R^{2}$ ranges from 0 to 1, where an $R^{2}$ equal to 0 means that the model cannot predict the target variable at all, and an $R^{2}$ equal to 1 means that the model can make a perfect prediction. $R^{2}$ can also have negative values, in which case the model’s prediction ability is not as good as calculating the mean of the target variable directly.

(2) Explainable Variance Score (EVar)

The explainable variance score measures the degree to which the dispersion of errors between all predicted and actual values is similar to the dispersion of the true values themselves.

(21) $$EVar=1-\frac{Var\left(y-\hat{y}\right)}{Var\left(y\right)}$$

A larger value of EVar indicates the better prediction ability of the model, and the best possible value is 1.

(3) Mean Absolute Error (MAE)

The mean absolute error is the expectation of the absolute value of the error between the predicted and actual values at each moment in time.

(22) $$MAE=\frac{1}{N}\overset{N}{\underset{i=1}{\sum }}\left|y_{i}-{\hat{y}}_{i}\right|$$

(4) Median Absolute Error (MedAE)

The median absolute error is the median of the absolute error of the predicted and actual values for all data points. The metric is robust to outliers.

(23) $$MedAE=median\left(\left|y_{1}-{\hat{y}}_{i}\right|,\cdots ,\left|y_{N}-{\hat{y}}_{i}\right|\right)$$

(5) Root Mean Square Error (RMSE)

The mean square error calculates the mean of the square of the error between the predicted and true values. The root mean square error, on the other hand, is the open square of the mean square error, which is consistent with the target variable in terms of magnitude.

(24) $$RMSE=\sqrt{\frac{1}{N}\overset{N}{\underset{i=1}{\sum }}{\left(y_{i}-{\hat{y}}_{i}\right)}^{2}}$$

### 4.6. Verification Experiment and Result Analysis

**Figure 12:**
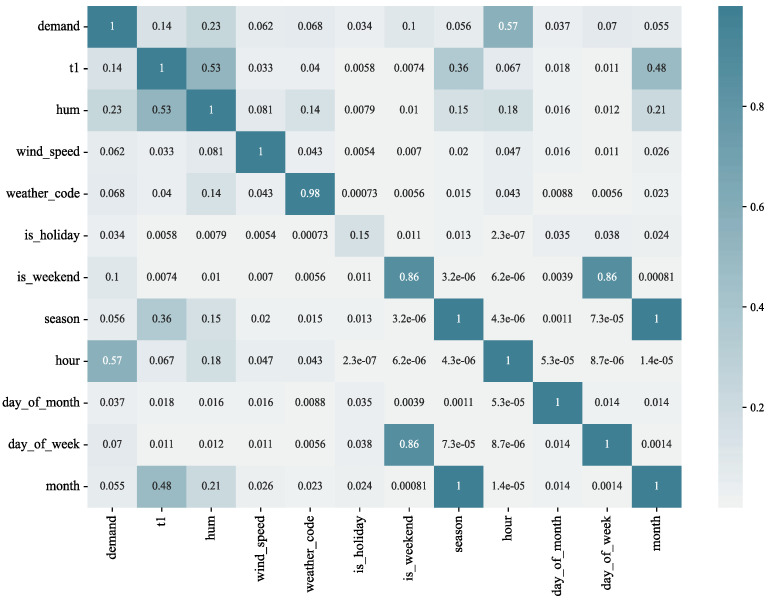
Heat map of MIC values between different variables.

To verify the validity of the proposed multivariate hybrid time series model, we conducted a validation experiment on the London area bike-sharing data set. The MIC method was first used for the variable selection part of this study, and the MIC values between the variables are shown in Figure 12.

The number of explanatory variables was studied in descending order according to the magnitude of MIC values between each explanatory variable and the dependent variable, and R2, EVar, MAE, and RMSE were used as measures.

**Figure 13:**
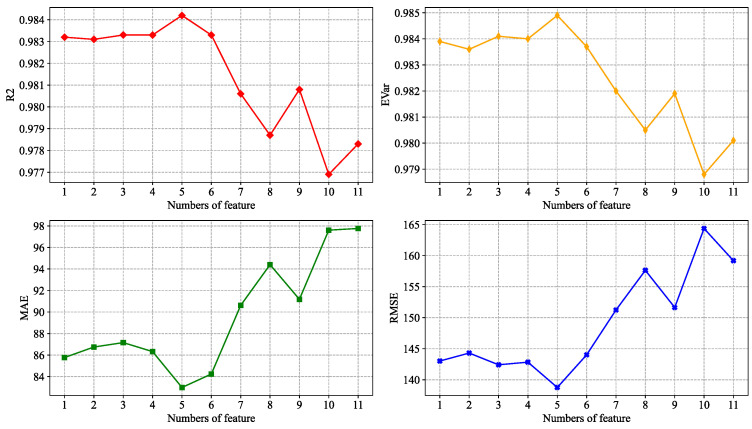
Comparison of the effects of models with different quantitative characteristics.

It can be seen from Figure 13 that the model works best when the number of features is 5. That is, the lowest feature selection threshold δ=0.07 and the combination of explanatory variables chosen is {hour, hum, t1, is_weekend, day_of_week}. It can be seen that the set of selected explanatory variables includes not only hour, weekend and day of week, which closely correspond to the morning and evening peaks of people commuting to work, but also includes the weather characteristics t1 and hum obtained by using Granger causality tests.

We performed a parameter search with the goal of the optimization of the effect of the hybrid model. The parameter search results of the TCN and GRU models are shown in Table 3 and Table 4.

We conducted two experiments: univariate prediction of the demand for bike-sharing and multivariate prediction of the demand for bike-sharing, respectively. Univariate prediction refers to the demand for bike-sharing as the only input without considering other explanatory variables. Multivariate forecasting, on the other hand, considers the influence of other explanatory variables on demand with the demand of bike-sharing as input, and obtains the corresponding explanatory variables through variable selection methods, which are also used as inputs to the model.

The comparison models used for the experiments include:

(1) Support Vector Regression (SVR) [51] (kernel = ‘rbf’, C = 1.0, max_iter = −1);
(2) XGBoost [52] (max_depth = 6, learning_rate = 0.1, eta = 1);
(3) ARIMA [53] (autocorrelation order: *p* = 9, difference order: d = 1, moving average orders: q = 0);
(4) ARIMAX (autocorrelation order: p = 9, difference order: d = 1, moving average orders: q = 8, exogenous variables: hour, hum, t1, is_weekend, day_of_week);
(5) LSTM (input_size = 6, hidden_size = 100, num_layers = 2, batch_size = 64, dropout = 0.2);
(6) History Average Model (HA) (history time step = 13);
(7) Prophet [54] (growth = “linear”, freq = ”H”, interval_width = 0.95);
(8) DeepAR [55] (input_size = 6, hidden_size = 64, num_layers = 3).

After averaging results over several iterations of the experiment, we determined the performance of each model on this dataset, and the specific evaluation metrics are shown in Table 5.

As can be seen from Table 5, the univariate model’s predictions are less effective overall than the multivariate model’s predictions, which indicates that the prediction performance of the model can be effectively improved with the inclusion of the selected explanatory variables; for example, the MAE and RMSE of the multivariate predictions are reduced by 7.0977 and 13.831, respectively, for the TCN-GRU model we used. In addition, some models such as DeepAR and Prophet may show non-adaptability to this dataset, and our experimental results are only better than those of the HA model. The hybrid model performs better than the single model in multivariate prediction, which proves that the hybrid model we use is more efficient and accurate based on the minimum sum of squares of errors.

**Figure 14:**
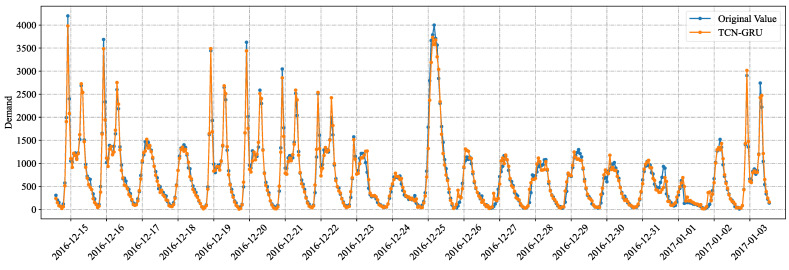
Fitting curve for bike-sharing demand data prediction.

The fit of our proposed multivariate TCN-GRU model to the actual values of bike-sharing demand for the last 480 data points (20 days) of the test set is shown in Figure 14.

## 5. Discussion

In recent years, bike-sharing has become an important way for people to travel in an environmentally conscious way. However, this free-form development mode has gradually revealed many problems, such as over-placement, the serious waste of public resources, and excessive growth, causing huge costs for urban management. The phenomenon of the indiscriminate parking of bike-sharing vehicles has led to a large number of public resources, such as subway station entrances, bus stops, bicycle lanes and pedestrian lanes, being occupied. The surge in the number of shared bicycles not only affects the cityscape, but also affects the safety of other public transportation. The uneven distribution of bicycles makes it difficult to meet the volatile users’ travel demands. These problems are new challenges for urban transportation managers.

**Figure 15:**
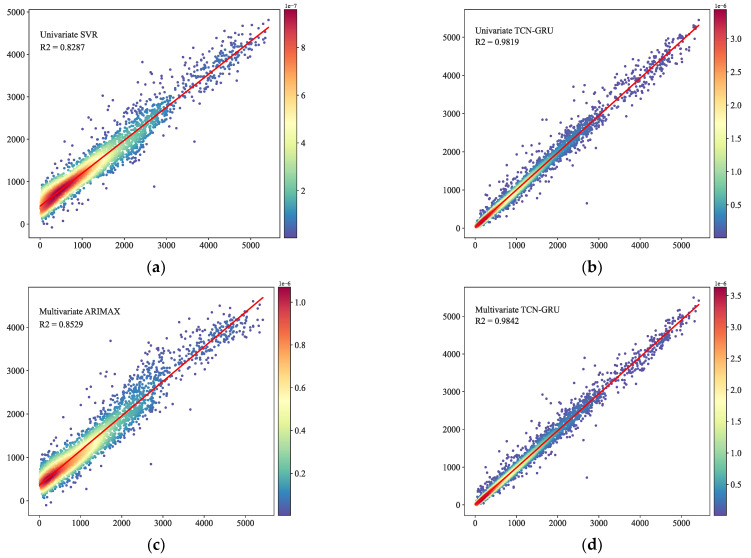
Model scatter density plot. (**a**) Univariate SVR scatter density plot. (**b**) Univariate TCN-GRU scatter density plot. (**c**) Multivariate ARIMAX scatter density plot. (**d**) Multivariate TCN-GRU scatter density plot.

To address the above problems, we took advantage of the fitting of deep learning models on nonlinear and nonsmooth sample data, and we used TCN and GRU models for bike-sharing demand prediction on the data set, combining the models with the principle of the minimum error sum of squares. The hybrid model improved the prediction accuracy, reduced the error, and effectively avoided the overfitting phenomenon. The experiments also proved that the models were less effective than multivariate prediction in the univariate prediction of bike-sharing demand, which meant that adding explanatory variables such as time, humidity, and temperature to the model input could improve the prediction effect. The R2 and EVar of the proposed multivariate TCN-GRU model in this paper were improved by at least 0.0023 and 0.0024, respectively, and the MedAE, MAE, and RMSE decreased by at least 2.7674, 7.026, and 10.55, respectively, compared with univariate forecasting models. At the same time, the R2 and EVar values of this model improved by at least 0.0009 and 0.0008, respectively, and the MedAE, MAE, and RMSE decreased by at least 0.4204, 1.6462, and 3.3241, respectively, compared with other multivariate forecasting models. In order to achieve a more intuitive comparison of the prediction accuracy, we drew a scatter density plot of the prediction effect of the compared models, as shown in Figure 15. In the comparison, we can see that the density distribution of the predicted values of the univariate SVR model, as well as the multivariate ARIMAX model, are not uniform, the distribution is relatively more dispersed, and the prediction effect is average. Our proposed multivariate TCN-GRU model predicts the values, while converging towards the actual values, and the fitting effect is better. Thus, we have established an efficient and robust short-term hybrid prediction model for bike-sharing demand considering multiple variables.

There are still several areas for improvement in this study.

(1) The combined model proposed in this paper showed good results in short-term bike-sharing demand prediction, and when we tried long-term prediction, the results were not satisfactory. Later, we will try to combine other models to improve performance in long-term prediction.
(2) In this study, we used a small-scale parameter-tuning method based on a grid search, and subsequently we considered other optimization algorithms for parameter searching which might improve the performance of the model.
(3) Due to limited data conditions, we were unable to obtain the main gathering locations of bike-sharing in the region, and thus could not extract spatial characteristics that could be used for further research following demand prediction.

## 6. Conclusions

In this paper, we built an accurate model that can be used for the short-term prediction of bike-sharing demand, using bike-sharing data from 2015 to 2017 in the London area. First, we analyzed multidimensional bike-sharing travel characteristics based on the explanatory variables such as weather, temperature, and humidity to understand the travel characteristics of local people, and thus facilitate traffic management and, to a certain extent, improve traffic congestion. Considering the nonlinear relationship between each explanatory variable and bike-sharing demand, we used the MIC method for variable selection, where variables were then used as part of the model input, and the experiments proved that adding explanatory variables could greatly improve the prediction performance of the model. In addition, considering the problems of over-fitting and poor stability that arise when using a single model on a small sample of data, we proposed a hybrid multivariate TCN-GRU model with the principle of the minimum error sum of squares, and the model showed strong efficiency and robustness. This can facilitate the accurate short-term prediction of bike-sharing demand in the region, which in turn provides decision support for intelligent dispatching and urban traffic safety improvements. It will also help to promote the development of green and low-carbon mobility in the future.

This study focuses on the possible prediction of factors affecting future bike-sharing in the London area by studying the time series data of bike-sharing traffic demand. Probably due to sensitivity issues, the data we obtained are limited, and we have been unable to obtain the actual locations of the main concentrations of shared bicycles, i.e., individual stations in the area. It would be useful to conduct a more in-depth study of intelligent scheduling, if the researchers can obtain the specific cluster locations of shared bikes in this area.

## Figures and Tables

**Table 1:** Data set fields description.

Field Name	Description	Example
timestamp	Timestamp for grouping data together	4 January 2015, 12:00
demand	Counting of new bike share	182
t1	Actual temperature (°C)	3.0
t2	Subjective perception of temperature (°C)	2.0
hum	Humidity percentage (%)	93.0
wind_speed	Wind speed value (km/h)	6.0
weather_code	Sunny: 1, Less Cloudy: 2, Cloudy: 3, Overcast:4, Rainy: 7, Storms: 10, Snowy: 26	3
is_holiday	Holiday: 1, Non-holiday: 0	0
is_weekend	Weekend: 1, Non-weekend: 0	1
season	Spring: 0; Summer: 1; Autumn: 2; Winter: 3	3
hour	24 h per day	12
day_of_month	Natural days per month	1
day_of_week	Monday: 0, …, Sunday: 6	1
month	January: 1, …, December: 12	6

**Table 2:** Results of causality tests for each variable.

Variable	Original Hypothesis	F-Statistic	Probability (p)
t1	t1 is not a bike-sharing demand Granger reason	230.8794	8.275 × 10^−8^
hum	hum is not a bike-sharing demand Granger reason	257.9023	1.296 × 10^−9^
weather_code	windspeed is not a bike-sharing demand Granger reason	20.1423	0.0728
wind_speed	weather code is not a bike-sharing demand Granger reason	5.1211	0.2036

**Table 3:** Parameter setting of the TCN model.

Parameter	Value
Time Steps	13
Nb_filters	64
Kernel_size	3
Nb_stacks	1
Epochs	80
Batch Size	32
Drop out	0.2
Dilations	[1, 2, 4, 8, 16, 32, 64]
Skip_connections	True
Kernel_initializer	he_normal
Optimizer	Adam
Activation Function	Rectified linear unit (ReLU)
Loss Function	Mean Squared Error (MSE)

**Table 4:** Parameters setting of GRU model.

Parameter	Value
Time Steps	13
Input Layer Units Number	100
Output Layer Units Number	1
Hide Layer Number	2
Hide Layer Units Number	100
Epochs	50
Batch Size	16
Learning Rate	0.001
Optimizer	Adam

**Table 5:** Prediction results of each model.

	Metrics	R2	EVar	MedAE	MAE	RMSE
Model						
Univariate	HA	0.4859	0.5234	457.8242	618.9324	864.8123
	Prophet	0.5971	0.6616	428.9174	504.2642	716.3489
	SVR	0.8287	0.8892	381.6209	308.4608	375.5922
	ARIMA	0.8379	0.8919	257.3966	297.9913	370.8495
	XGBoost	0.9657	0.9669	383.0468	111.5021	205.4212
	LSTM	0.9730	0.9748	315.4528	112.4182	178.9126
	GRU	0.9767	0.9769	312.3578	112.8749	171.3778
	TCN	0.9806	0.9813	288.7231	89.8644	154.1625
	TCN-LSTM	0.9808	0.9817	50.5265	90.0193	152.5853
	TCN-GRU	0.9819	0.9825	52.1868	90.0910	149.3043
Multivariate	DeepAR	0.7278	0.7861	401.2352	456.8923	613.7432
	ARIMAX	0.8529	0.8990	250.8287	285.9122	358.4603
	TCN	0.9829	0.9837	49.1962	86.2586	143.8991
	GRU	0.9817	0.9813	72.7963	104.2761	154.4806
	LSTM	0.9799	0.9807	61.567	98.7257	156.6573
	TCN-LSTM	0.9833	0.9841	48.1795	84.6395	142.0784
	TCN-GRU	0.9842	0.9849	47.7591	82.9933	138.7543

## Data Availability

Publicly available dataset was analyzed in this study. It can be found here: https://www.kaggle.com/hmavrodiev/london-bike-sharing-dataset (accessed on 1 May 2022).
